# Supplementary material for: Postsynaptic Neuroligin-1 Mediates Presynaptic Endocytosis During Neuronal Activity
Source: Front Mol Neurosci. 2021 Oct 8;14:744845. doi: 10.3389/fnmol.2021.744845 (PMC8531268; doi:10.3389/fnmol.2021.744845)
Supplement: Supplementary file 1 [file Data_Sheet_1.pdf]

Postsynaptic neuroligin-1 mediates presynaptic endocytosis during neuronal activity

Jiaqi Keith Luo, Holly Melland, Jess Nithianantharajah, Sarah L Gordon

Supplementary Methods

Quantifying size of synaptic vesicle pools

To quantify the size of synaptic vesicle pools, the readily releasable and reserve pool were released sequentially by two separate trains of stimuli. Neurons were stimulated in the presence of 1 µM bafilomycin A1 with a train of 60 action potentials delivered at 30 Hz (100 mA, 1ms pulse width) to trigger fusion of the readily releasable pool, followed by 1200 action potentials at 10Hz to mobilise the reserve pool. Fluorescence change from baseline (ΔF) was calculated as (F – F0)/F0, where F0 is the baseline fluorescence prior to stimulation. ΔF is then normalised to the maximum (i.e. total) fluorescence change during alkaline imaging buffer perfusion ($\Delta F/F_{total}$). The maximum $\Delta F/F_{total}$ after the termination of the first stimulation and before the commencement of the second stimulation was used to report the size of the readily releasable pool. The maximum $\Delta F/F_{total}$ after the termination of the second stimulation and before alkaline imaging buffer perfusion was used to report the size of the total recycling pool. The size the reserve pool was calculated as total recycling pool – readily releasable pool.

Monitoring dynamic cycling (exo- and endocytosis) of synaptic vesicles

Neurons were stimulated with a train of 300 action potentials at 10 Hz (100 mA, 1ms pulse width), then imaged for a further 150 seconds to capture post-stimulus compensatory endocytosis (images captured at 2s intervals). Baseline subtracted fluorescence ΔF was normalised to maximum fluorescence revealed by NH_4 to obtain $\Delta F/F_{total}$. The peak of $\Delta F/F_{total}$ during the stimulus train was taken as the stimulation peak. $\Delta F/F_{total}$ after the termination of stimulation and before perfusion with alkaline imaging buffer was fitted with the function:

$$\Delta F/F_{total} = ae^{-\frac{t}{\tau}} + platea$$

The parameter τ represents the endocytic time constant.

Quantifying exocytic rate and the size of the total recycling pool

Neurons were stimulated with a train of 1200 action potentials at 10Hz (100 mA, 1ms pulse width) in the presence of 1 µM bafilomycin A1 to inhibit reacidification of synaptic vesicles. To estimate exocytic rate, ΔF was normalised to the peak fluorescence increase during stimulation ($F_{peak)}$) to obtain $\Delta F/F_{peak}$ and fitted with the function:

$$\Delta F/F_{peak} = 1 - e^{-\frac{t}{\tau}} + F_0$$

The parameter τ represents exocytic time constant.

Early exocytic rate was estimated by fitting the $\Delta F/F_{peak}$ of the first five frames after stimulation commenced with the linear function:

$$\Delta F/F_{peak} = kt + F0$$

The parameter k represents early (linear) exocytic rate.

The maximum $\Delta F/F_{total}$ during the last 20 seconds (10 frames) prior to perfusion of alkaline imaging buffer expressed as a percentage was used quantify the size of the total recycling pool in this experiment.

Statistical analyses for individual synapses

Quantification of fluorescence imaging for individual synapses was identical to coverslip-average data. Differences between WT and *Nlgn1*$^{-/-}$ synapses and their confidence intervals were estimated using a two-level mixed-effects linear model with normally distributed varying intercepts assigned to each coverslip and the cluster robust error estimator. This ensures we obtain reliable mean and error estimates in the presence of correlation among synapses from the same coverslip as well as non-constant error across coverslips. Exocytic/endocytic time constants and linear exocytic rates were log-transformed prior to the analysis.

We tested for multimodality using Hartigan's dip test in each dataset for each genotype as evidence for the presence of heterogenous synapse sub-populations of significant size. We found no significant evidence to reject a unimodal distribution for all datasets.

Supplementary data

SUPP FIG 1

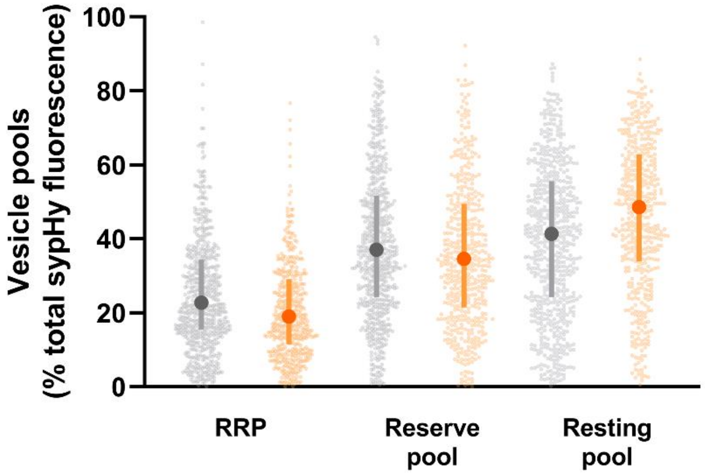

Supplementary Figure 1: Lack of Nlgn1 does not impact the size of synaptic vesicle pools. *Nlgn1$^{-/-}$* and WT cultured neurons were transfected with sypHy. Neurons were stimulated with 60 action potentials (AP) at 30Hz to trigger fusion of the readily releasable pool (RRP), followed by 1200 AP at 10Hz to mobilise the reserve pool of vesicles (together composing the total recycling pool), in the presence of bafilomycin to inhibit vesicle reacidification. The remaining vesicles, which do not undergo fusion, comprise the resting pool. Fluorescence change was monitored over time, and neurons were then perfused with NH_4Cl to reveal total sypHy fluorescence; fluorescence change from baseline was normalised to the total sypHy fluorescence. Scatter plot shows size of distinct vesicle pools (readily releasable, reserve and resting as a proportion of total sypHy fluorescence) for individual *Nlgn1$^{-/-}$* and WT synapses. n = 1434 - 1926 synapses from 10 – 13 individual fields of view from independent coverslips (pool size x genotype interaction p = 0.056). Individual synapse data points are presented with median and interquartile range.

SUPP FIG 2

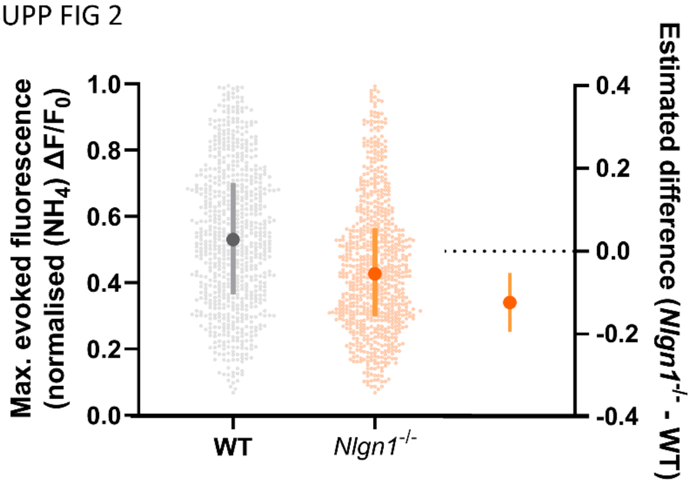

Supplementary Figure 2: *Nlgn1$^{-/-}$* synapses display a change in the balance of exocytosis and endocytosis. *Nlgn1$^{-/-}$* and WT neurons transfected with sypHy were stimulated with 300 action potentials at 10Hz, and change in fluorescence monitored over time. Neurons were then perfused with NH_4Cl to reveal total sypHy fluorescence. Scatter plot shows peak sypHy fluorescence during stimulus train, normalized to total fluorescence, of individual *Nlgn1$^{-/-}$* and WT synapses. Scatter plot of 733 – 758 synapses from 14-15 individual fields of view from independent coverslips are presented with median and interquartile range. Forest plot shows estimated difference between groups with 95% CI (p = 0.001).

SUPP FIG 3

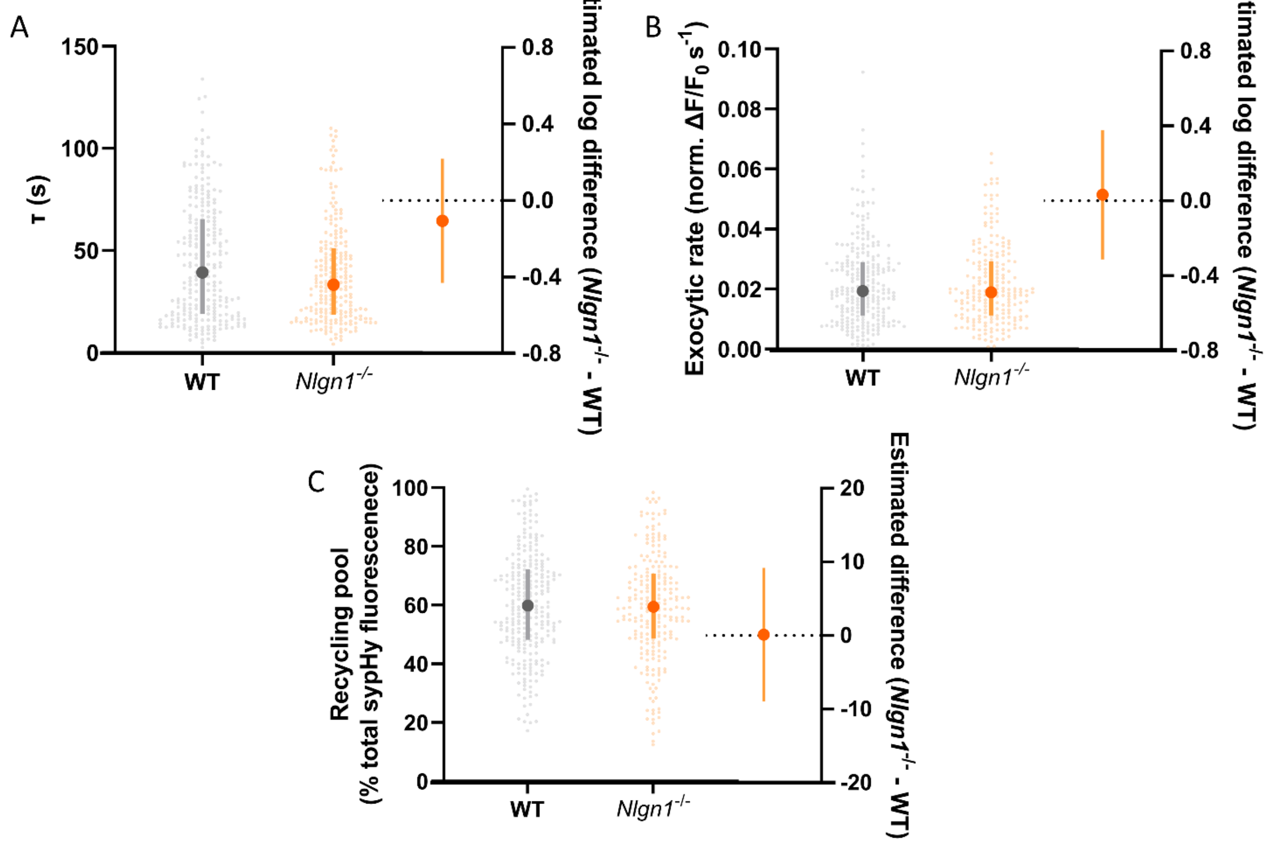

Supplementary Figure 3: Rate of exocytosis is unchanged in *Nlgn1*$^{-/-}$ synapses. *Nlgn1*$^{-/-}$ and WT neurons transfected with sypHy were stimulated with 1200 AP (10Hz) in the presence of bafilomycin. Scatter plots show data of 224 - 260 individual synapses from 8-9 individual fields of view from independent coverslips with median and interquartile range. Forest plots show estimated difference between genotypes with 95% CI. **A**: Exocytic time constant (tau, s) and estimated difference between groups on log-transformed data (p = 0.523). **B:** Linear rate of exocytosis ($\Delta F/F_0$ s^{-1}) as assessed by linear fit over first 10 s of stimulation and estimated difference between groups on log-transformed data (p = 0.853). **C:** Size of total recycling pool assessed as peak fluorescence during stimulus train, as a % of total sypHy fluorescence revealed by NH_4Cl (p = 0.982).

SUPP FIG 4

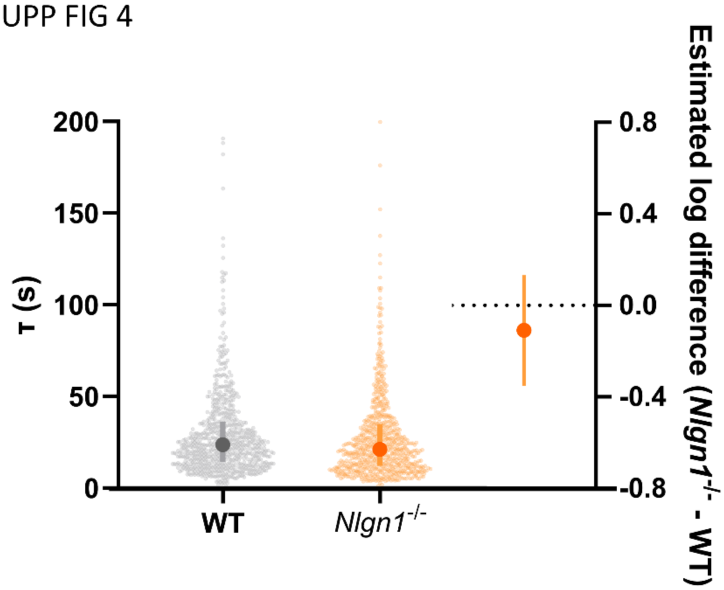

Supplementary Figure 4: Loss of *Nlgn1$^{-/-}$* does not impact post-stimulus compensatory endocytosis. *Nlgn1$^{-/-}$* and WT neurons transfected with sypHy were stimulated with 300 action potentials at 10Hz, and change in fluorescence monitored over time. Scatter plot shows endocytic decay time constant (tau, s) from single exponential decay curve fit to data following end of stimulus train (from fig 2A) of 733 – 758 individual synapses from 14-15 individual fields of view from independent coverslips, as in Supp Figure 2. Individual synapse data points are presented with median and interquartile range. Forest plot shows estimated difference between groups on log-transformed data with 95% CI (p = 0.351).
